# Duke Surgery Research Central: an open-source Web application for the improvement of compliance with research regulation  

**DOI:** 10.1186/1472-6947-6-32

**Published:** 2006-07-27

**Authors:** Ricardo Pietrobon, Anand Shah, Paul Kuo, Matthew Harker, Mariana McCready, Christeen Butler, Henrique Martins, CT Moorman, Danny O Jacobs

**Affiliations:** 1 Center for Excellence in Surgical Outcomes, Division of Orthopaedic Surgery, Duke University Medical Center, Box 3094, Durham, NC, 27710, USA; 2 School of Medicine, University of Pennsylvania, 3450 Hamilton Walk, Philadelphia, PA, 19104, USA; 3 Department of Surgery, Duke University Medical Center, Box 3704, Durham, NC, 27710, USA

## Abstract

**Background:**

Although regulatory compliance in academic research is enforced by law to ensure high quality and safety to participants, its implementation is frequently hindered by cost and logistical barriers. In order to decrease these barriers, we have developed a Web-based application, Duke Surgery Research Central (DSRC), to monitor and streamline the regulatory research process.

**Results:**

The main objective of DSRC is to streamline regulatory research processes. The application was built using a combination of paper prototyping for system requirements and Java as the primary language for the application, in conjunction with the Model-View-Controller design model. The researcher interface was designed for simplicity so that it could be used by individuals with different computer literacy levels. Analogously, the administrator interface was designed with functionality as its primary goal. DSRC facilitates the exchange of regulatory documents between researchers and research administrators, allowing for tasks to be tracked and documents to be stored in a Web environment accessible from an Intranet. Usability was evaluated using formal usability tests and field observations. Formal usability results demonstrated that DSRC presented good speed, was easy to learn and use, had a functionality that was easily understandable, and a navigation that was intuitive. Additional features implemented upon request by initial users included: extensive variable categorization (in contrast with data capture using free text), searching capabilities to improve how research administrators could search an extensive number of researcher names, warning messages before critical tasks were performed (such as deleting a task), and confirmatory e-mails for critical tasks (such as completing a regulatory task).

**Conclusion:**

The current version of DSRC was shown to have excellent overall usability properties in handling research regulatory issues. It is hoped that its release as an open-source application will promote improved and streamlined regulatory processes for individual academic centers as well as larger research networks.

## Background

Research is of paramount importance to accomplish the institutional and educational mission of academic medical centers. Frequently, however, researchers implement their investigational protocols without fully understanding regulatory requirements and restrictions, ultimately failing to minimize the risk of liability and regulatory scrutiny [1-4]. As a consequence, well-publicized regulatory flaws in the implementation of research studies have raised public questions about the integrity of the entire research process. These breaches in trust are harmful, since public confidence in the integrity of research is critical not only for funding support among constituents and participation in clinical trials, but, importantly, also for confidence in the interventions that result from trials themselves. Frequent lawsuits are related to regulatory issues, the most common including: lack of appropriate informed consent, fraud on the Food and Drug Administration (FDA) regulations, violation of the common rule, or breach of an agreement to abide by the Belmont Report [5,6]. Other adverse consequences of non-compliance for faculty members include suspension of their projects, intensive investigations, and future suspension of rights for the involved faculty member or institution to apply for future federally supported research funds[7].

Although necessary for maintaining the integrity of research, regulatory compliance involves significant costs for academic centers [8-10]. Recent research has demonstrated that up to 32% of the total research time of investigators was devoted to nonclinical activities such as Institutional Review Board submission [11]. As expected, this high percentage results in major expenditures, with direct variable costs reaching up to somewhere around $2,000 devoted to nonclinical costs per subject enrolled in industry-sponsored trials [11]. Thus, the costs associated with regulatory compliance are substantial. In addition to high cost, implementation of regulatory compliance is also made difficult by frequent changes in the healthcare regulatory environment. Traditionally, this uncertainty has added to the perception of compliance activities as an invasive disruption of research activities whenever an extra effort is required to achieve regulatory compliance.

The logistical and cost issues could be improved by centralized Web-based systems, allowing research administrators to have increased assurance about their ability to reach acceptable levels of regulatory compliance within their institutions. Previous efforts designed to ensure widespread compliance have fallen short on multiple aspects, including lack of adequate oversight, bureaucracy without enough operational support from academic centers to facilitate researcher's work, and a time strain on research administrators.

In the early 1990's, the National Library of Medicine (NLM) provided support for a large number of universities to develop the Integrated Academic Information Management System (IAMS). This system sought to provide individuals with information in an organized format, therefore reducing the burden associated with the handling of multiple documents.

As an example, the University of Cincinnati has developed the eGrants system, which aimed at the full digitization of the pre-award and post-award phases, thus achieving compliance throughout all phases of the grant lifecycle. In addition, it was expected that eGrants would streamline and reduce errors in the grant preparation, routing, and submittal process, ultimately improving its quality and consistency, as well as reducing time and cost spent with research management activities [12-14]. The initial outcomes of the program included a substantial increase in intramurally funded projects, a 200% increase in funds for educational research from local sources other than the medical school, and two new grants funded from extramural sources. This program provided means to monitor faculty progress, thus providing objective data to facilitate the promotion process [15]. eGrants has now been replaced by a more comprehensive set of tools [13]. Despite significant advances demonstrated by the University of Cincinnati, the system is not freely available and thus cannot be implemented at other centers. Other institutions have implemented variations of IAMSs, but not exclusively for research support and not as comprehensive as eGrants [16].

We sought to design a Web-based application to streamline the regulatory process, both for investigators and administrators. The primary objective was to provide both sets of users an interface that provides up-to-date regulatory information and task scheduling, in an environment that would promote efficient and secure communication between users. We describe Duke Surgery Research Central (DSRC), a Web application designed to streamline the research administration process and improve regulatory compliance in academic centers.

## Implementation

### Goals

The objective of the DSRC project was to ensure regulatory compliance in research activities involving regulatory documentation for Institutional Review Board (IRB) submission, grant proposals, and contracts. To improve regulatory compliance, the system should a) allow researchers to visualize the administrative progress of their projects; b) allow research administrators to track all projects, and c) generate reports that would establish the current compliance status of individual researchers. Ultimately, this system should be able to minimize researchers' time in circulating regulatory documents and obtaining necessary signatures.

### Design objectives

The decision to build a customized application was made after we searched, without success, for cost-effective available business tools and other systems available from academic institutions that would be able to handle our regulatory needs. Objectives were defined based on direct requirements from personnel directly involved with research administration of our institution, as well as security recommendations from Health Insurance Portability and Accountability Act (HIPAA) guidelines. Our search resulted in the definition of technical requirements describing the optimal characteristics of the final application:

1. Researcher interface should be simple and intuitive in order to meet the requirements of users with different levels of computer literacy.

2. The application should run on an Intranet within the campus firewall to enhance protection against security breaches.

3. Researchers should be informed about the existence of new tasks assigned to them through a variety of mechanisms, including e-mail and RSS (real simple syndication) feeds.

4. Tasks assigned to users should have a clear sequence of activities to be followed by the researcher. At a minimum, they should have instructions on how to complete the regulatory activity, provide instructions and electronic templates for that task, provide an interface for document submission, and allow users to inquire research administrators about the status of their administrative tasks currently in-process.

5. The application should store additional information that might be of value to researchers, including links with information about research opportunities, tools to search for available number of patients for prospective research, and other applications that will change the image of a regulatory site from a burden into a service to the researcher.

6. Research administrators should have the ability to track the times of completion of researchers stratified by department, project name, leader, task, deadline date, research administrator in charge, and principal investigator

7. Research administrators should be able to delegate tasks that are commonly associated grouped in "bundles." By grouping tasks that can be assigned through a single command, task bundles are time efficient for research administrators.

### Software architecture

**Figure 1:**
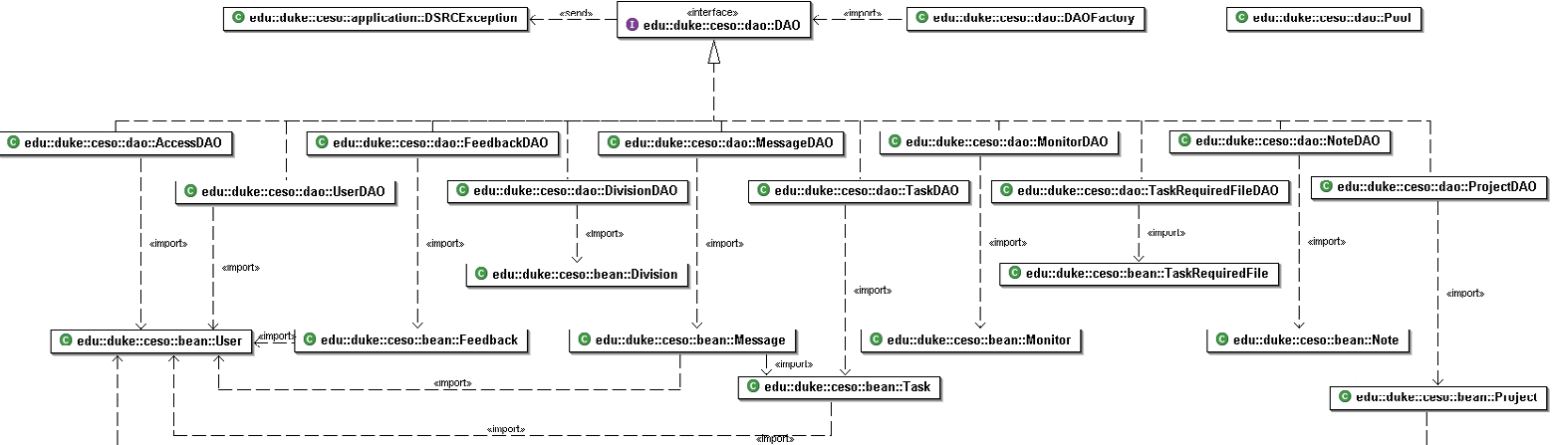
UML Class Diagram that implements the Data Access Object design pattern.

**Figure 2:**
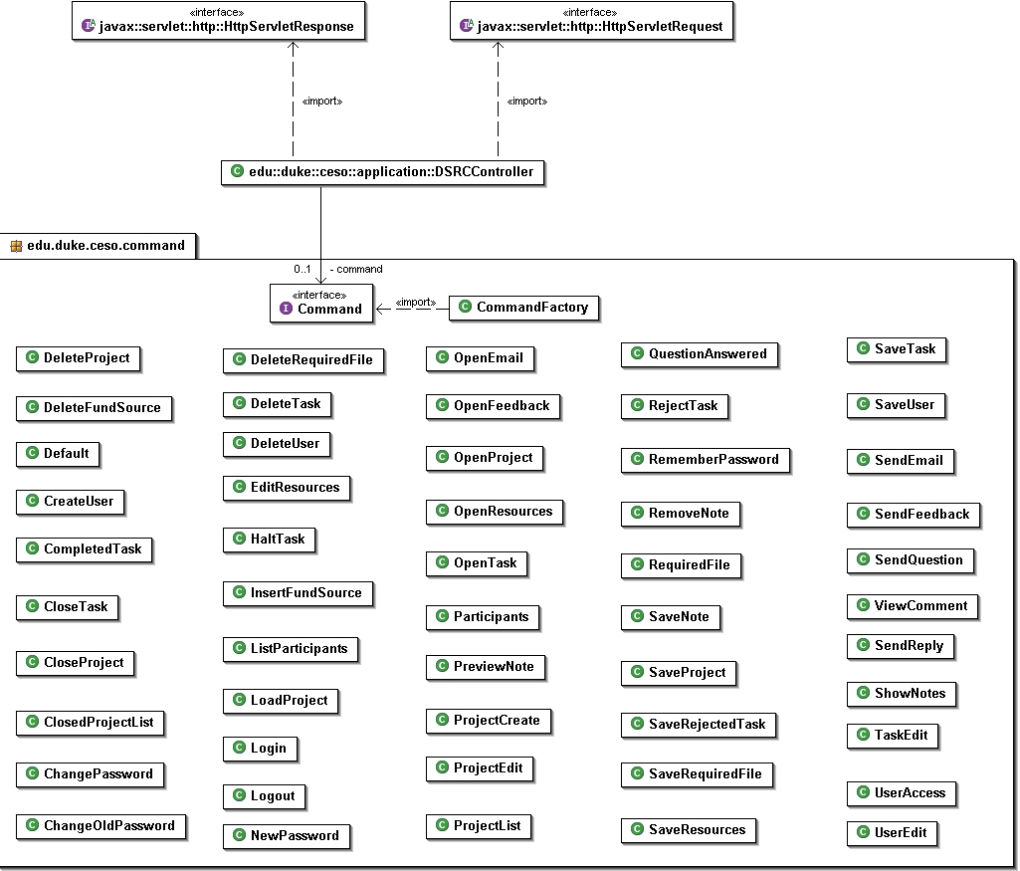
UML Class Diagram that implements the Command design pattern.

**Figure 3:**
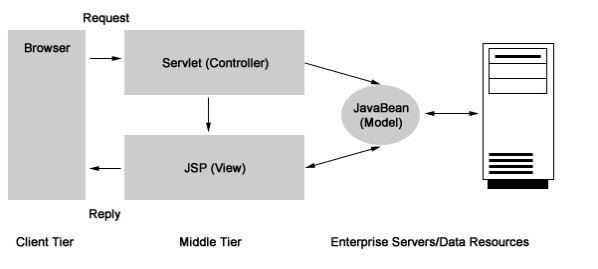
Relation between Model and View.

The class component model is displayed in Figures 1 and 2. The software was developed using Java as the programming language and the Model-View-Controller (MVC) as its design model. The concept of MVC design model is that an application consists of three components: a central Model, Views that represent the model to the user, and Controllers of the Model. In our application, the Model contains the logic displayed by the application, including database access, numeric algorithms, and algorithms for data manipulation. In contrast, the View and Controller components represent the interface of the application. Stated in a different manner, the controller is an input component that supplies information to the Model, while the View is an output component displaying information from the Model [see Figure 3].

Figure 1 depicts the UML Class Diagram that implements the Data Access Object (DAO) design pattern. This solution was used to abstract and encapsulate all access to the data source. The DAO manages the connection with the data source to obtain and store data.

Figure 2 illustrates the UML Class Diagram that implements the Command design pattern. This pattern takes the load from the Controller (MVC) component by implementing each command on a different component. This component must implement an Execute method, so, instead of the Controller having to know how to route requests from the View, it needs only to call the Command object's Execute method.

### Interface for DSRC

Interface design was developed using a combination of paper prototyping [17] and use cases. The interface for the Duke Surgery Research Central can be divided into screens for researchers and research administrators.

### Researchers

**Figure 4:**
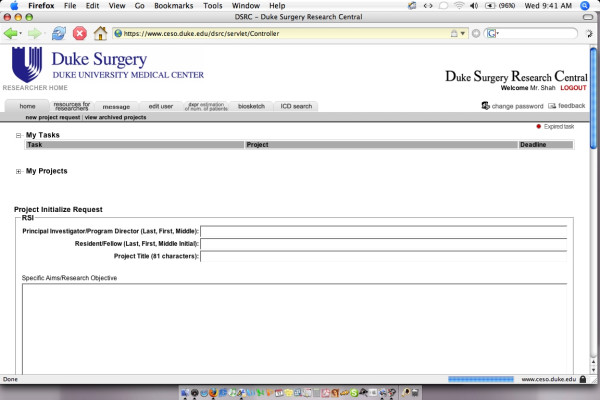
Researcher Interface.

The interface for researchers provides functionality for each of the following activities [see Figure 4]:

1. View a complete list of research projects in which the researcher is currently working within DSRC

2. Within each project, view a time line of all associated tasks. These tasks include not only active tasks but also previously completed tasks. Files can be retrieved from previously completed tasks, thus constituting a storage location for all study-related files.

3. Receive instructions for completion of each task, also having the ability to ask questions to research administrators for instructions that have not been clearly given. For each assigned task, researchers can: simply mark a task as completed (for tasks not requiring the submission of a file); download necessary templates and submit them; or fill documents out on an online environment.

4. External to the completion of specific regulatory tasks, researchers also have links for commonly used resources for research such as templates for regulatory documents, information on grant opportunities, and applications that allow for determination of number of patients in the Department of Surgery with a specified combination of diagnostic and procedure codes.

### Research administrators

**Figure 5:**
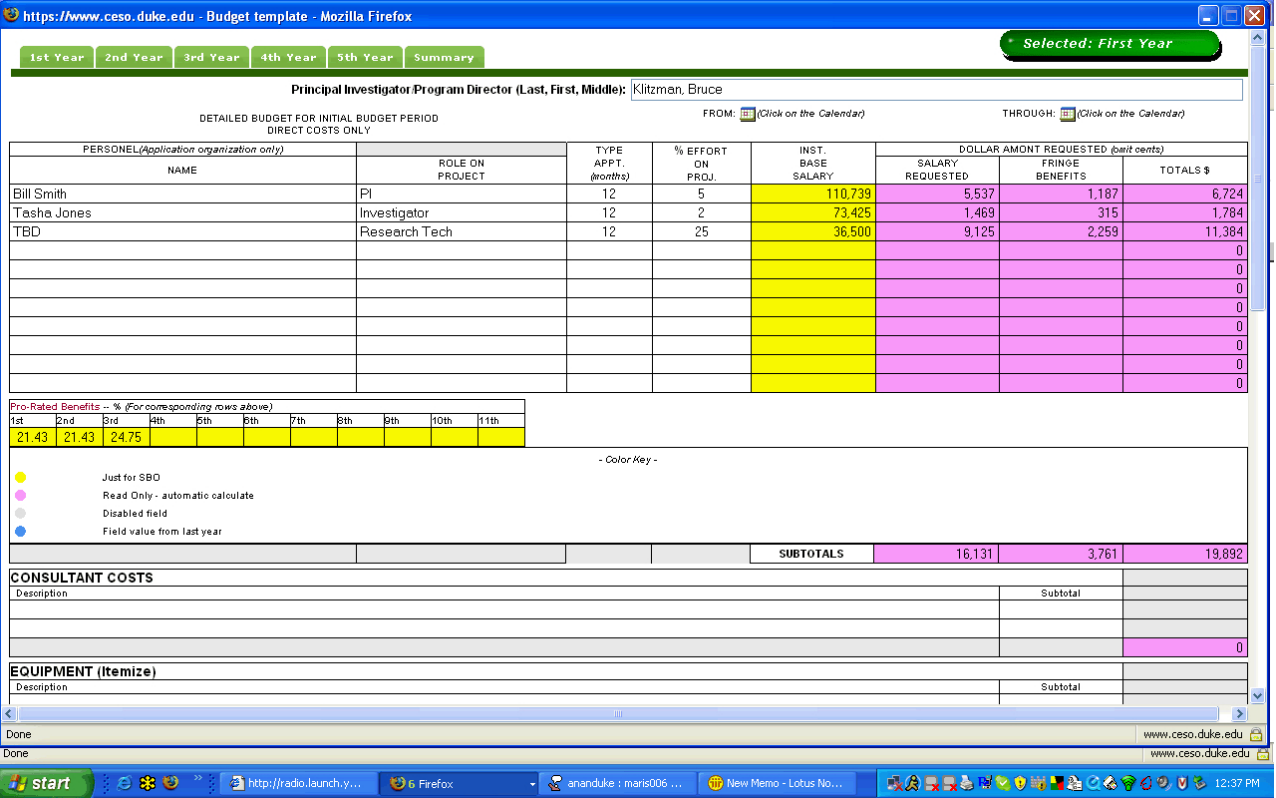
Administrator Interface.

The interface for research administrators allows for the following functionality [see Figure 5]:

1. Register and update new users with descriptive information such as department affiliation.

2. Create new projects using information provided by researcher for grants, IRB protocols, and contracts that will be submitted to the research administration office

3. Create new tasks within each project, providing individual researchers with instructions, templates, and deadlines for submission of necessary documentation

4. Download files submitted by researchers containing the information assigned in their task

5. Read and respond to questions submitted by researchers

6. Modify project and task information when necessary

7. View complete list of projects being processed in the system at any given time, sorted by project name, leader, task, deadline, research administrator name, or researcher name

8. In order to ensure that tasks assigned to researchers have been accessed, research administrators have access to a list of researchers who have not accessed the system after seven days since the date of assignment. In this way, research administrators can be notified in circumstances where a researcher might be away and not able to complete the assigned task or if a researcher new to the system may not feel comfortable accessing the application, all without risking that the task might not be completed

9. Access and update additional resources for researchers external to individual regulatory tasks (see description in section describing the interface for researchers).

### Usability evaluation

Usability was evaluated by formal usability analysis and field observation levels. Formal usability analysis was conducted by an external consultant to avoid bias from authors participating in the development of the DSRC application. The evaluation included ten different users with no previous experience with the application. User selection was based on matching of educational characteristics compatible with our target users, including a degree in a health care field.

Formal usability tests followed a protocol where users were observed by two evaluators and had to complete assigned regulatory tasks. Users were able to ask questions at any point in time. The following application factors were evaluated: speed, easy of learning, easy of using, understanding of functionality, and navigation. We also made notes about number of errors made while using the application. Each participant answered a questionnaire at the end of the formal usability analysis with items about interface problems, missing features, and suggestions for overall improvement.

Field observations were conducted by one of the co-authors (MM) and comprised observation of researchers and research administrators during the execution of common regulatory activities. Meetings were held with investigators and research administrators in order to obtain their feedback in terms of usability issues, graphical design, and difficulties that were addressed during the initial four months of deployment at our institution. In contrast with the evaluation performed during the formal usability tests, the evaluator (MM) restricted her notes to material that could be communicated back to our programmer (HM) for immediate implementation.

## Results

### Researchers

When using DSRC, researchers start by notifying research administrators via email or phone about a project that they would like to initiate. This project can be an IRB protocol, grant, or industry contract. Research administrators then create a new project in DSRC, providing password-protected access to the research team involved with the project. All project access is limited to participants and restricted to the campus Intranet for security compliance. Once the project is created, researchers receive an email notifying them about the creation of the new project and their respective tasks. Each task is assigned to individual investigators in the project team. Although single tasks are frequently performed by more than one project member, from a project management perspective, we felt that it is more reliable to make individual members accountable. Individual researchers can then log in to the DSRC site to verify the nature of their tasks, download necessary templates for task completion, and upload all required files or completed the forms online. As soon as the researcher completes the required task, research administrators are notified and will carry the administrative process further. Researchers also have the ability to securely send questions to research administrators concerning individual tasks as well as to access all files related to previously completed tasks. This iterative process is repeated as many times as necessary until all necessary documents associated with the project are satisfactorily completed.

### Research administrators

After receiving an email notice from the researcher, research administrators will capture all available information about the project so that it can initialized in DSRC. This information includes project names, participant names and contact information, as well as information on any regulatory documents available for the project. This information will be available to researchers at any point in time in the project page. Once a project is created, corresponding tasks are created and assigned to individual researchers, also including the name of the research administrator in charge of verifying submitted documents and answer any task-related questions. For projects with tasks that are frequently grouped, administrators have the ability to create "task bundles" that can be assigned all at once. Bundling saves time in project set up and also ensures that all required tasks are consistently included in the target project.

Three types of tasks can be created: (1) Tasks that require researchers to simply check when an activity is accomplished (e.g., attending a regulatory research training session), (2) Tasks that require the download of a file that is completed and then submitted back in DSRC, and (3) tasks where forms can be completed online. Every time a task is created, DSRC sends an automatic email to researchers notifying them that they have been assigned to a task. When researchers fail to access the task within seven days form the day of assignment, research administrators are notified. Actions can then be taken to identify the reason for lack of access, including technical problems preventing their access to DSRC, which are then appropriately corrected. When researchers complete a task, the initial page for research administrators will automatically display a task requesting them to verify the completed task and also to create the subsequent tasks when necessary. Finally, research administrators have access to a master list containing all projects assigned to themselves, being able to sort them by investigator, date of completion, status (expired vs. non-expire), and completion status (completed vs. non-completed).

### Usability

#### 1. Formal usability

Formal usability results demonstrated that, from the perspective of researchers, the DSRC application presented good speed, was easy to learn and use, had a functionality that was easily understandable, and a navigation that was intuitive [see Table 1]. Only 2 users had errors during their formal usability sessions, both being related to not being able to browse and find the files within their computers. These errors were considered to be associated with computer literacy rather than with the design of DSRC itself. No further features were requested during the formal usability analysis phase.

**Table 1:** Formal Usability

DSRC speed is excellent.	Strongly disagree	0/10
	Disagree	0/10
	Neutral	0/10
	Agree	2/10
	Strongly agree	8/10
		
DSRC is extremely easy to learn	Strongly disagree	0/10
	Disagree	0/10
	Neutral	1/10
	Agree	2/10
	Strongly agree	7/10
		
DSRC is extremely easy to use	Strongly disagree	0/10
	Disagree	0/10
	Neutral	0/10
	Agree	2/10
	Strongly agree	8/10
		
It is very easy to understand all functionality available within DSRC (e.g., download files, upload files, etc)	Strongly disagree	0/10
	Disagree	0/10
	Neutral	1/10
	Agree	2/10
	Strongly agree	7/10
		
The navigation in DSRC is highly intuitive	Strongly disagree	0/10
	Disagree	0/10
	Neutral	0/10
	Agree	1/10
	Strongly agree	9/10

#### 2. Field usability

The first two months of field usability measurement were primarily focused on fixing software bugs related to minor problems in coding, including buttons that were not working appropriately, navigational issues related to users being taken to the wrong page after completing a task, and the implementation of a consistent interface across all pages of the application. Of importance, the initial development phases relied heavily on feedback from research administrators rather than extensive testing by software developers. This process allowed for immediate feedback in early stages of the project, when fundamental issues of the application were raised by research administrators.

After the initial bugs were eliminated, issues raised by field testers encompassed three major themes [see Table 2]. First, in the creation of projects, variable categorization was preferred over free text. Research administrators felt that discrete (categorized) variables were not only able to generate better reliability across users, but also to improve the ability to search for information. Also in creation of projects phase, research administrators felt the need to have searching boxes that allowed them to quickly find researcher names, which is in contrast with the previous use of drop boxes. Second, when deleting any tasks, research administrators requested more explicit warning messages asking whether they were sure about their action. Explicit messages ensured that the probability of a mistaken deletion would be minimized. Finally, researchers felt that messages reassuring them that a certain task had been completed should be a constant feature throughout all pages, even when it represented redundancy. For example, in the initial application design a task would simply be shown as completed after a file had been submitted. For the purposes of additional feedback, researchers felt that receiving an e-mail confirming that the task had indeed been completed would be helpful as a reassurance, also providing them with a written record of their completion.

**Table 2:** Field Usability Issues

**Creation of projects (research administrator)** "Create discrete categories for all variables, e.g., funding mechanism" "Create search mechanism to go through hundreds of user names once the system is fully implemented"
**Creation of new tasks (research administrator)** "When deleting a task, create clear messages warning users before action is taken"

**Task completion (researcher)** "When researchers complete a task, an email is sent to them so that they can have a "proof" of having completed it."

### Comparison to existing software applications

Of note, ProIRB (ProIRB Plus, St Petersburg, FL), CyberIRB (ProIRB Plus, St Petersburg, FL), IRB+ (West Beach Software, Santa Barbara, CA), and Click Commerce eResearch Portal (Click Commerce Inc., Chicago, IL) are among several existing commercial software applications that aim to streamline the research regulatory process for academic centers. The first program utilizes a Microsoft Access-based database to manage IRB-related data, while the latter three provide the institutional user with customizable forms and Web-based IRB research data management. Similar to DSRC, each of these programs provides interfaces for both researchers and IRB administrators. However, DSRC provides a unique interface for researchers, as they are not only able to complete regulatory tasks, but also search for related grant and funding opportunities from a single portal. Similarly, administrators are able to access and add to functions outside of regulatory tasks, such as providing researchers with information regarding funding, recently published research, and IRB and bioethics tutorials; these functions are customizable according to individual departmental needs. We feel that compared to existing applications, DSRC provides a significantly improved interface for administrators, allowing them not only to manage workflow, but also use DSRC as a medium for communication for researchers.

Importantly, in contrast to the mentioned commercial packages, DSRC is freely available to all users. As research regulations and methods continue to evolve, the authors intend to continue to modify and distribute the source code freely to the general public. One perceived limitation of DSRC might be reliance on internal institutional information technology (IT) departments for technical support and source code modification (i.e. customization), compared to commercial software developers who provide varying levels of technical support for their own programs. The authors believe that, in the long-run, academic institutions can derive cost savings from having the ability to freely modify DSRC to their specific departmental and workflow needs.

## Discussion

We have described the design, implementation and testing of Duke Surgery Research Central, an open source Web tool developed to facilitate and improve regulatory compliance in academic research settings. Our formal usability results have found that researchers were satisfied with the overall easy to use and functionality of the application, not requiring any major additions to the initial design. During field usability, we found that categorization, searching capabilities, warning messages, and reassurance about the execution of tasks were helpful for users to understand the consequences of their actions while using the application.

The major challenge during the development of DSRC was the wide variability in degree of computer literacy by our researchers. Although one would initially expect that researchers are most often computer literate, in agreement to other studies, we identified multiple issues that were related to the lack of basic knowledge on how to use a computer [18,19], such as Internet navigation using a Web-browser and use of drop-down menus in DSRC. The same difficulties were not faced by research administrators, who not only were familiar with the use of computers for their daily work activities, but who also spend more time using the application and thus tend to be more proficient despite the higher complexity of their interface.

In order to minimize the difficulties with usability issues, we recommend software development in multiple waves. By exposing a progressively larger number of users to the application, modifications and additions to the software can be made at each step, thus decreasing the number of issues encountered by users with lower levels of computer literacy. Finally, it is important that the initial versions of the application have a reduced number of features, with further implementation of functionality being determined according to researchers' needs during field testing.

We believe that one of the major reasons for success of this project was the time and effort spent in the acquisition of requirements established by research administrators and investigators. This process established not only the basis for the application functionality, but also provided a sense of ownership by both groups. Since this application becomes part of their daily activities, it is important that researchers and research administrators feel that their input was taken into account and that the application accurately represents their needs. In our project, we have accomplished the acquisition of application requirements through the use of paper prototyping [17] and obtained feedback through constant contact between programmers and end-user mediated by a business analyst familiar with the research process (MM).

### Current utilization

Currently, DSRC is used exclusively at the Department of Surgery at Duke University, but the source code is being freely distributed as an incentive to promote use at other institutions. The design of DSRC was motivated by the large volume of regulatory documentation handled by the Surgery Business Office, including industry and government-sponsored studies. As a strategy for integration into the department, DSRC was initially introduced among selected faculty members who were identified as more likely to provide the development team with feedback. This initial implementation was followed by access to resident physicians submitting smaller grant proposals that involved relatively less involved regulatory process. In the final stage, faculty members and research staff in all divisions were involved. Although its use is currently voluntary within the Department of Surgery (as DSRC is a developing beta technology), it is hoped that DSRC will soon become the standard within our Department and others divisions at our institution. Moreover, global implementation of DSRC at our institution will streamline and create one standard for the workflow at our institutional IRB. It should be noted that DSRC, in its current version, can most readily be implemented by institutions that have an academic IT department to assist in modifying source code for institution-specific uses.

### Features for future versions

Desirable features for future versions include an increase in the integration among DSRC and other applications previously developed by our group [20,21] to streamline the research process. We believe that the provision of additional tools might modify the traditional view of regulatory compliance perceived as a burden to an image of support for researchers' activities. Other features include the addition of features that allow for internal audits within DSRC, such as data extraction regarding time spent in each of the research administration activities. These data can then be used by those evaluating the research process in conducting conduct cost-effectiveness studies on potential cost savings associated with the system.

## Conclusion

The current version of DSRC was shown to have excellent overall usability properties in handling research regulatory issues for both researchers and administrators. It is hoped that its release as an open-source application will promote improved and streamlined regulatory processes for individual academic centers as well as larger research networks.

## Availability and requirements

Project Name: Duke Surgery Research Central

Project Home Page:  (click link for "Free software")

Operating System: Linux/Windows

Programming Language: Java

Other requirements: Tomcat 5.x

License: GNU General Public License

Any restrictions to use by non-academics: none

## Abbreviations

DSRC – Duke Surgery Research Central

GNU – GNU's Not Unix (recursive acronym)

## Competing interests

The author(s) declare that they have no competing interests.

## Authors' contributions

All authors read and approved the final manuscript.

RP – assisted in the design of the application, field tests for detection of bugs, design and analysis of usability tests, and manuscript draft.

AS – assisted in the design of the application, field tests for bug detection, and revision of manuscript draft

PK – assisted in the design of the application, field tests for bug detection, and revision of manuscript draft

MH – assisted in the design of the application, field tests for bug detection, and revision of manuscript draft

MM – assisted in the design of the application, field tests for bug detection, and revision of manuscript draft

CB – assisted in the design of the application, field tests for bug detection, and revision of manuscript draft

HM – assisted in the design of the application, wrote source code, and revised manuscript draft

CTM – assisted in the design of the application, field tests for bug detection, and revised manuscript draft

DOJ – assisted in the design of the application, field tests for bug detection, and revision of manuscript draft

## Pre-publication history

The pre-publication history for this paper can be accessed here:


